# Optimal Battery Sizing in Photovoltaic Based Distributed Generation Using Enhanced Opposition-Based Firefly Algorithm for Voltage Rise Mitigation

**DOI:** 10.1155/2014/752096

**Published:** 2014-06-19

**Authors:** Ling Ai Wong, Hussain Shareef, Azah Mohamed, Ahmad Asrul Ibrahim

**Affiliations:** Faculty of Electrical Engineering and Built Environment, Universiti Kebangsaan Malaysia, 43600 Bangi, Malaysia

## Abstract

This paper presents the application of enhanced opposition-based firefly algorithm in obtaining the optimal battery energy storage systems (BESS) sizing in photovoltaic generation integrated radial distribution network in order to mitigate the voltage rise problem. Initially, the performance of the original firefly algorithm is enhanced by utilizing the opposition-based learning and introducing inertia weight. After evaluating the performance of the enhanced opposition-based firefly algorithm (EOFA) with fifteen benchmark functions, it is then adopted to determine the optimal size for BESS. Two optimization processes are conducted where the first optimization aims to obtain the optimal battery output power on hourly basis and the second optimization aims to obtain the optimal BESS capacity by considering the state of charge constraint of BESS. The effectiveness of the proposed method is validated by applying the algorithm to the 69-bus distribution system and by comparing the performance of EOFA with conventional firefly algorithm and gravitational search algorithm. Results show that EOFA has the best performance comparatively in terms of mitigating the voltage rise problem.

## 1. Introduction

Recently, distributed generation (DG) with green energy sources such as photovoltaic (PV) generation has drawn a lot of attention worldwide since they are clean, environment-friendly, and reliable. However, there are some issues to be resolved before the installation of PV-based DG (PVDG) in the distribution networks due to the intermittent power output from PV systems. The PVDG is usually uncontrolled and it depends greatly on solar radiation. The amount of power generation increases or decreases irrespective of the load demand at a particular time; instead it is depending upon the availability of solar energy. Therefore, voltage fluctuation problem occurs when the load demand is not in line with the amount of power generated from PVDG. Sudden voltage rise or voltage drop in the network can create undesired damages which can be costly to the users. Therefore, some solutions have been proposed in the literature to alleviate voltage fluctuation problem. A method using load control to regulate voltage on DG embedded network is proposed by Scott et al. [1]. Besides, voltage regulation of PV generator can also be controlled by introducing a series reactor in the service line [2]. Meanwhile, battery energy storage system (BESS) can be one of the good options in mitigating the voltage rise problem [3, 4]. Nevertheless, high installation cost is required for BESS installation at every bus in the network. Thus it is crucial to obtain the optimal location for BESS in the system.

Obtaining the optimal size for BESS is also important since the bigger the capacity of the BESS, the more it costs. Optimal sizing of BESS helps to obtain a suitable BESS size for the system to maintain voltage regulation. In the literature, different approaches have been developed in determining optimal size for BESS. Shen [5] obtained the optimal combined size for PV and BESS for standalone PV system by calculating the loss of power supply probability for different size combinations of PV and BESS. Meanwhile, the chance constrained programming approach and the concept of design space were used by Arun et al. [6]. Here, a sizing curve that relates both PV rating and the corresponding minimum BESS capacities was plotted. Brekken et al. [7] proposed sizing and control methodologies for zinc-bromine BESS. Khatib et al. [8] utilized the MATLAB curve fitting tool to fit the sizing curve obtained from a standalone PV system energy flow and then derived a formula for optimal sizing of PV and BESS. Furthermore, Ru et al. [9] determined the BESS size for grid-connected PV system by optimizing the BESS cost and the net power purchase from the grid.

In recent years, heuristic optimization techniques have gained a lot of attention from researchers due to their better performance compared to mathematical optimization techniques in coping with large and complex optimization problems. There are different types of heuristic optimization techniques. One of the early techniques was genetic algorithm (GA) [10], followed by other techniques such as ant colony optimization (ACO) [11], particle swarm optimization (PSO) [12], artificial bee colony algorithm (ABC) [13], gravitational search algorithm (GSA) [14], firefly algorithm (FA) [15], artificial plant optimization algorithm (APOA) [16, 17], artificial physics optimization (APO) [18], shuffled frog leaping algorithm (SFLA) [19], and membrane computing [20]. Vrettos and Papathanassiou [21] applied GA in optimizing the size of the hybrid system consisting of wind turbines, PV, and BESS system. The multiple-objective function in this work minimizes the generation cost and maximizes the renewable energy source penetration. Chen et al. [22] also applied GA to calculate the optimal energy storage size by optimizing the investment cost model which is a nonlinear objective function. Apart from GA, the ABC algorithm was used to obtain the optimal BESS capacity in order to mitigate the voltage rise problem in the PV embedded distribution network [23].

However, metaheuristic optimization algorithms have the problem of being trapped in local optimum and slow convergence rates due to their random searching process. This leads to the development of hybrid algorithms that can overcome these issues effectively. FA is chosen for the optimization process in this current study since it is relatively simple and easy to implement. However, like most of the metaheuristic optimization algorithms, FA also has its own disadvantages. In order to further improve the performance of original FA in terms of convergence rate, the opposition-based learning [24] is integrated into FA while the idea of inertia weight FA [25] is also incorporated at the same time to improve the ability of FA to escape from local optimum. In this paper, enhanced opposition-based firefly algorithm (EOFA) is proposed to determine the optimal size of BESS in a PVDG integrated radial distribution network for mitigating the voltage rise problem.

## 2. Formulation of Optimization Problem

As mentioned earlier, the root cause of voltage rise in a PVGD integrated system is the intermittent nature of power or current injections at PVDG bus. This problem can be solved by using BESS as it has the ability to act as a power source or sink with the help of its bidirectional power converter. The BESS will operate in charging mode and as a current sink if there is excess output power from PVDG while it will discharge and act as a current source if more power is needed to maintain the voltage profile of the system. The BESS is set to be charged when the PVDG is active, while, at night when PVDG is idle, the BESS will discharge to a certain preset state of charge. In this study, PVDG is modeled as a current source while BESS is modeled as a current source or sink. EOFA is first used to obtain the optimal average hourly BESS active output power for the PVDG integrated system. In this optimization, the solution set (searching agent) is the BESS power while the purpose of the optimization is to minimize the voltage deviation of the PVDG bus by using the optimal BESS power value in order to maintain the voltage within 0.95 pu to 1.05 pu range. Therefore, the objective function, *f*_1_, can be expressed as

(1) $$\begin{array}{c} f_{1}=\min\{\begin{array}{cc} \left|V_{i}\left(t\right)-1.05\right|, & \text{if}\;\;V_{i}\left(t\right)>1.05\\ \left|V_{i}\left(t\right)-0.95\right|, & \text{if}\;\;V_{i}\left(t\right)<0.95, \end{array} \end{array}$$

where *V*_*i*_(*t*) is the per unit (pu) value for voltage at bus *i* at hour *t*. In this study it is assumed that the voltages are constant at a particular hour of the day. However, it can be extended for shorter intervals for more accurate results.

After the optimal BESS power values for each hour are obtained, the state of charge (SOC) of the BESS for each hour can be calculated as [26]

(2) $$\begin{array}{c} \text{SOC}=100\left(1-\frac{\int I_{\text{bs}}dt}{Q}\right), \end{array}$$

where *I*_bs_ is the current for the BESS, *t* is the time in hour, and *Q* is the BESS capacity in Ampere hour (Ah). When the SOC reaches its maximum limit (SOC_max⁡_) or minimum limit (SOC_min⁡_), the BESS will be turned off temporarily until it charges or discharges again. Considering the SOC, optimal size or capacity of the BESS can be decided again by using the optimization algorithm. The performance of BESS increases when the number of BESS inactive hours due to the SOC constraint is minimized. In this optimization, the solution set is the possible solution for BESS capacity. The optimal BESS capacity should give the minimum number of off-time or inactive hours. Considering this criterion the second objective function, *f*_2_, to obtain the size of the BESS can be defined as

(3) $$\begin{array}{c} f_{2}=\min\left(N_{\text{idle}}^{\text{bs}}\right), \end{array}$$

where *N*_idle_^bs^ is the total number of time BESS is turned off when SOC reaches either SOC_max⁡_ or SOC_min⁡_.

## 3. Enhanced Opposition-Based Firefly Algorithm (EOFA)

### 3.1. Overview of Original FA

FA is a heuristic optimization algorithm based on the flashing characteristics of fireflies [15]. The main functions of the flashes are to attract the mating partners as well as to attract the potential prey. FA is illustrated based on three rules where, firstly, all fireflies are of the same sex and thus the attraction between fireflies is independent regardless of their sex. Secondly, the attraction is proportional to the brightness of the fireflies and it decreases when the distance between the fireflies increases. In other words, the brighter fireflies will attract the less bright ones. The fireflies will move randomly if all of them have the same brightness. Thirdly, the brightness of the fireflies is decided by the landscape of the objective function.

**Figure 1:**
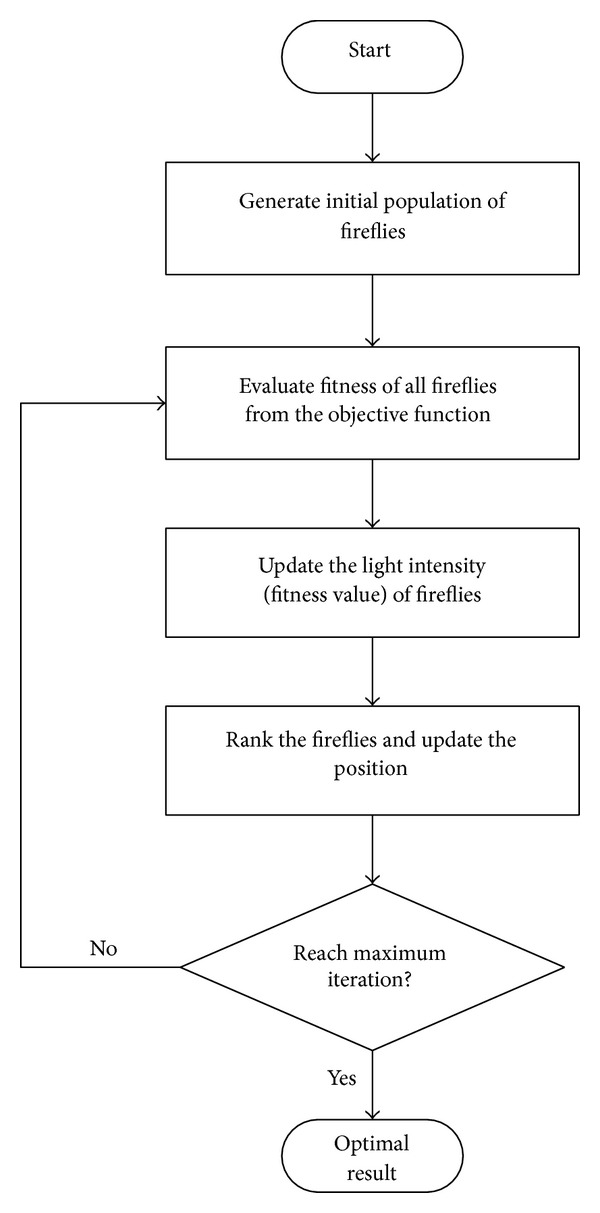
Flowchart for FA.

Two main parts in FA are the variation of light intensity and the attractiveness between the fireflies. The attractiveness of the fireflies is affected by the light intensity (brightness) which then is related to the objective function. The attractiveness *β*(*r*) of a firefly can be defined as [15]

(4) $$\begin{array}{c} \beta \left(r\right)=\beta_{o}e^{-\gamma r^{2}}, \end{array}$$

where *β*_*o*_ is the attractiveness at *r* = 0, *γ* is the light absorption coefficient, and *r* is the Cartesian distance between two fireflies as shown in [15]

(5) $$\begin{array}{c} r_{ij}=||x_{i}-x_{j}||=\sqrt{\overset{d}{\underset{k=1}{\sum }}{\left(x_{i,k}-x_{j,k}\right)}^{2}}, \end{array}$$

where *i* and *j* represent two different fireflies at *x*_*i*_ and *x*_*j*_ while *x*_*i*,*k*_ is the *k*th component of the spatial coordinate *x*_*i*_ of *i*th firefly. Meanwhile, the movement of the firefly *i* which is attracted by the brighter firefly *j* is defined in [15]

(6) $$\begin{array}{c} x_{i}=x_{i}+\beta_{o}e^{-\gamma r^{2}}\left(x_{j}-x_{i}\right)+\text{alpha}\left(\text{rand}-\frac{1}{2}\right), \end{array}$$

where the second term is due to the attraction and the third term is due to the randomization. In the third term, alpha is the randomization parameter while rand is the random number generator uniformly distributed between zero and one. In each following iteration, alpha decreases with a decreasing factor, delta, as shown in (7). The flowchart for FA is shown in Figure 1:

(7) $$\begin{array}{c} \text{alpha}\left(t+1\right)=\text{alpha}\left(t\right)\times \text{delta},\;0<\text{delta}<1. \end{array}$$

FA can perform better if it is compared to other algorithms as particle swarm optimization (PSO) and genetic algorithm (GA) in terms of efficiency and successful rate [27]. However, the performance of FA can become less satisfied when the dimension of search space increases. Therefore, EOFA is introduced to further improve the performance of FA where FA is integrated together with the inertia weight function [25] and opposition-based learning [24].

### 3.2. Techniques for Improving Original FA

#### 3.2.1. Opposition-Based Learning

Opposition-based learning was suggested by Tizhoosh [24] and it has been employed in several heuristic optimization algorithms such as genetic algorithm [24], differential evolution algorithm [28], ant colony optimization [29], and gravitational search algorithm [30] in order to enhance the performance of these algorithms. Basically, optimization process such as FA always starts with an initial population (solutions) which is created randomly due to the absence of a priori information about the solutions. Then the algorithm will try to search for the best solutions. However, there can be a possibility that the initial guess for the solutions is far away from the actual solutions. The convergence rate can be improved when the initial guess is closer to the actual solutions. The chance to start with the solutions closer to the optimal value can be increased by obtaining the opposite set of solutions simultaneously. The set of population that is closer to the optimal value will be chosen as initial population. The similar method can be adopted as well for each solution in the current population. The concept of opposite number is demonstrated below.

Let *x* ∈ *R* be a real number within a defined interval where *x* ∈ [*a*, *b*]. The opposite number *x*_*o*_ can be defined as shown in

(8) $$\begin{array}{c} x_{o}=a+b-x. \end{array}$$

Similarly, this concept can be extended to the case with higher dimensions. Let *P*(*x*_1_, *x*_2_,…, *x*_*m*_) be a set of points in *m* dimensional search space where *x*_*i*_ ∈ [*a*_*i*_, *b*_*i*_] and *x*_1_, *x*_2_,…, *x*_*m*_ ∈ *R*. Then the points in the opposition set *P*_*o*_(*x*_*o*1_, *x*_*o*2_,…, *x*_*om*_) can be defined as shown in

(9) $$\begin{array}{c} x_{oi}=a_{i}+b_{i}-x_{i},\;i=1,2,\ldots ,m. \end{array}$$

By using the definition for opposite number, the opposition-based optimization can be developed as follows. Let *P*(*x*_1_, *x*_2_,…, *x*_*m*_) be the set of points in *m* dimensions search space which is the candidate solution for an optimization problem. According to opposition theorem, *P*_*o*_(*x*_*o*1_, *x*_*o*2_,…, *x*_*om*_) will be the opposition set for *P*(*x*_1_, *x*_2_ …, *x*_*m*_). Suppose that *f*(*x*) is the function used to measure the performance of candidate solution; thus if *f*(*P*) is greater than or equal to *f*(*P*_*o*_), then a set of points in *P* can be replaced by *P*_*o*_ or else *P* is maintained.

#### 3.2.2. Inertia Weight

Inertia weight-based FA was proposed by Tian et al. [25] where an inertia weight function as shown in (10) is applied to (6):

(10) $$\begin{array}{c} \omega \left(t\right)=\omega_{\max}-\left(\omega_{\max}-\omega_{\min}\right)*\left(\frac{t}{\text{Maxgeneration}}\right), \end{array}$$

where *ω*(*t*) is the inertia weight at time *t*, *ω*_max⁡_ and *ω*_min⁡_ are the initial and final values of the inertia weight, respectively, throughout the iteration process, *t* is the current iteration, and Maxgeneration is the maximum number of iterations as defined in the initialization process of FA. The inertia weight function decreases linearly with respect to time where, at the beginning stages, large inertia weight increases the global exploration ability and thus prevents the algorithm from being trapped in local optima. At the end of the stages, the reduced inertia weight enhances the local exploration of the solutions.

The movement of the firefly to update its position using inertia weight-based FA can be illustrated as shown in

(11) xi(t)=ω(t)xi(t)+βoe−γrij2(xj(t)−xi(t))+alpha(rand−12).

The incorporation of opposition-based learning and inertia weight-based function in FA is to avoid premature convergence as well as to enhance the searching ability of the algorithm where the global exploration at the beginning of the optimization process and the local exploration at the end of the optimization process are improved.

### 3.3. EOFA

**Figure 2:**
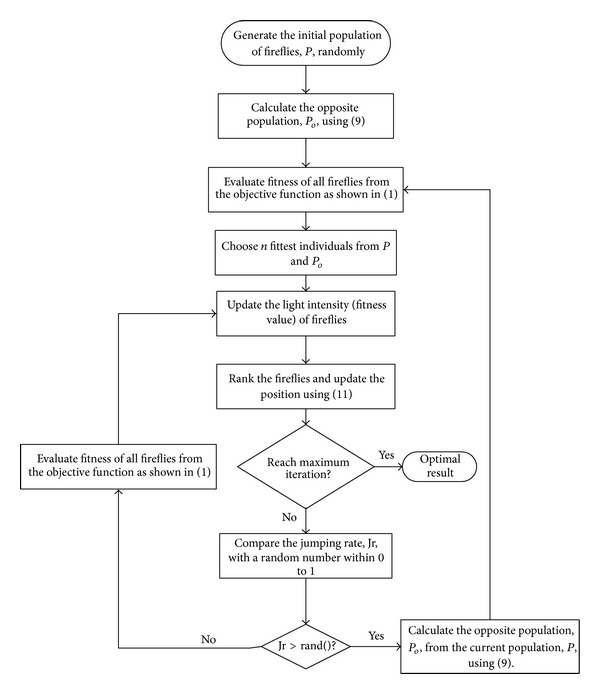
Flowchart for EOFA.

Opposition-based population initialization and opposition-based steps for EOFA with the population size of *n* and dimension of *m* are shown in Figure 2. For the initialization, the initial population of fireflies, *P*, is generated randomly, and then the opposite population, *P*_*o*_, is calculated using (9). The *n* fittest fireflies are chosen from *P* and *P*_*o*_ to become the first population in opposition-based optimization process.

In EOFA, each firefly updates the light intensity (fitness value) after the evaluation of the fitness from the objective function. Then the fireflies rank and update their positions using (11). In EOFA, a jumping rate, Jr, is used to decide if the opposite population is generated or not according to (12). If Jr is greater than the generated random number, the opposite population is generated and the next population contains the *n* fittest individuals chosen from currents *P* and *P*_*o*_ or else the next population remains as the current population, and *P* is generated from the update of firefly's position. The optimization process repeats until the criteria given are met, where in this case it is the maximum number of iterations:

(12) $$\begin{array}{c} \text{generation}\;\;\text{of}\;\;\text{opposite}\;\;\text{population}=\{\begin{array}{cc} \text{yes}, & \text{if}\;\;\text{Jr}>\text{rand}\left(\right)\\ \text{no}, & \text{otherwise}. \end{array} \end{array}$$

The opposition-based optimization enables the algorithm to search for the global optimum points in a faster way. The superior performance of EOFA in escaping from the local optimum points as well as the higher convergence rate is shown in the results section. The steps and implementation of EOFA in mitigating voltage rise problem are discussed in the following section.

## 4. Implementation of EOFA in Mitigating Voltage Rise Problem

In order to mitigate the voltage rise problem, a BESS that helps to control the suitable amount of power available in the grid is needed. At the same time, the optimal size of the BESS can be determined by EOFA using the following steps.

- Generate the initial population *P* randomly with a population size *n*. Each firefly consists of the information of the BESS active power output value for each hour.
- Calculate the opposite population *P*_*o*_ using (9) and choose only *n* fittest firefly individuals from *P* and *P*_*o*_.
- Run the load flow program for the system under study with a PVDG and BESS. The *n* fittest individuals are evaluated in the load flow according to the objective function as shown in (1) for charging and discharging, respectively.
- Update the light intensity (fitness value) of the firefly and then rank and update the position of the firefly using (11).
- Check the stopping criteria where, in this case, it is the maximum number of iterations. If the maximum number of iterations is not achieved yet, compare the jumping rate (Jr) based on the criteria given by (12). If the opposite generation is generated, again, only *n* fittest current firefly individuals from *P* and *P*_*o*_ are chosen for next iteration.
- Repeat step (iii) until stopping criteria are achieved. The BESS output values for each hour are obtained.
- Then EOFA is used again to determine the optimal BESS size by considering the SOC constraints using the objective function as shown in (3).

## 5. Results and Discussion

### 5.1. Performance Assessment of the Proposed EOFA

Fifteen benchmark test functions for unconstrained global optimization [31] are chosen in order to evaluate the performance of EOFA. The name, dimension size, and the global minima of each test function are presented in Table 1. Besides, a comparative study is conducted with gravitational search algorithm (GSA) [14] in order to show the superior performance of EOFA in solving most of the benchmark optimization problems. In addition, FA [15] is included in the comparison as well showing the improvement of conventional method by using EOFA. The setting of parameters including population size, the number of maximum iterations, and some parameters from the optimization algorithms is decided through trial and error procedure and experimentation depends on the system size, the complexity of the objective functions, and convergence characteristics of the optimization algorithms as well as the time consumed to complete the optimization process. In this work, the population size, *n*, is set to be 50 and the number of maximum iterations is taken as 1000 for all algorithms used in the comparison. For FA and EOFA, the values for *β*_*o*_, initial alpha, delta, and gamma are defined as 1, 0.2, 0.97, and 1, respectively. For EOFA, the jumping rate is Jr = 0.3, while the inertia weights, *ω*_max⁡_ and *ω*_min⁡_, are 1.4 and 0.5, respectively. For GSA, the initial gravity constant, *G*_*o*_, is set to be 100 while the best applying force, Kbest, decreases monotonically from 100% to 2.5%. The parameter *τ* is set to be 8% of the total number of dimensions.

**Figure 3:**
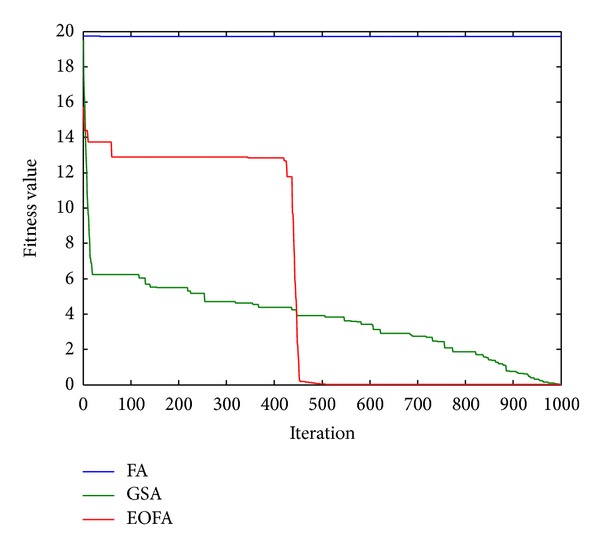
Comparison of performance of FA, GSA, and EOFA for benchmark function F1 with dimension size 30.

**Figure 4:**
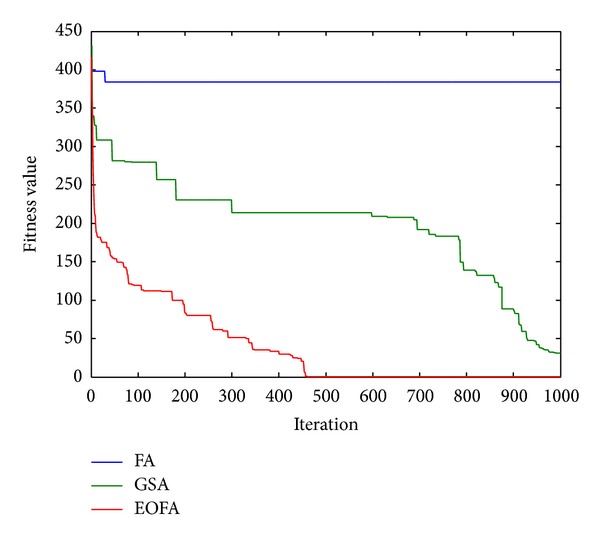
Comparison of performance of FA, GSA, and EOFA for benchmark function F10 with dimension size 30.

After 50 runs on each test function, the performances (fitness value) of each algorithm are reported in Table 2 where the values with “∗” indicate the best performance and the values with “∗∗” indicate the worst performance. It can be seen from Table 2 that the performances for FA are the worst most of the time compared to GSA and EOFA. This can be caused by premature convergence after trapping in a local optimum. On the other hand, it can be observed that EOFA has the best performance for most of the test functions except for F2, F7, and F11 where GSA outperforms EOFA. It is known from the reviews that different algorithms may perform better than others for different problems [32, 33]. Performances in terms of convergence between FA, GSA, and EOFA for randomly chosen functions are illustrated in Figures 3 and 4. It can be seen from the figures that FA always converges prematurely and exhibits an unsatisfied result. Meanwhile, both GSA and EOFA are able to escape from local minima and provide better results. However, EOFA has the higher convergence rate and gives better results compared to GSA.

### 5.2. Performance of EOFA in Voltage Rise Mitigation

**Figure 5:**
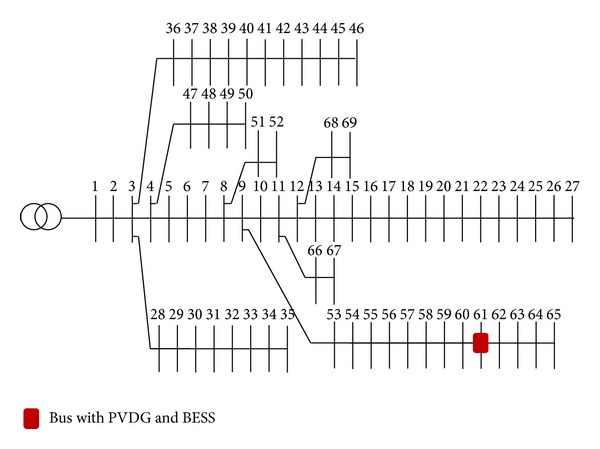
Single-line diagram of the 69-bus distribution system.

**Figure 6:**
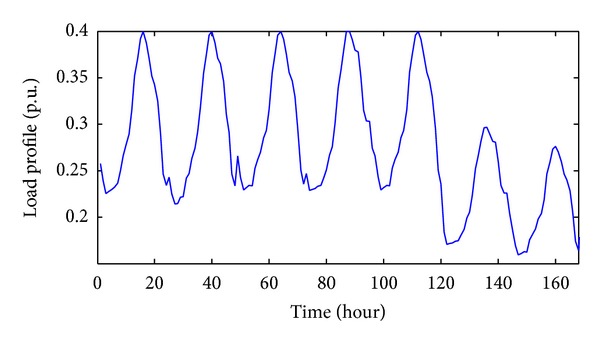
Hourly individual load profile for one week.

In this work, the 69-radial-bus system as shown in Figure 5 is used where a 3.66 MW PVDG is installed at Bus 61. The system data can be obtained from [34]. The pattern for PVDG output power values is obtained from the output of a lower scale grid connected PVDG system installed at the Faculty of Engineering and Built Environment, Universiti Kebangsaan Malaysia. In this study, the hourly PVDG output power from 9 am to 6 pm collected for three months (91 days) is used. Besides, BESS is assumed to be installed at the PVDG bus. According to the PVDG bus voltage at a particular hour, if the voltage exceeds the maximum limit (1.05 pu) or is lower than the minimum limit (0.95 pu), the BESS will be activated and the BESS power for that particular hour is decided by the optimization process either to inject (discharge) or to store (charging) power from the system. The upper and lower limits for the SOC of the BESS are set to be 100% and 20%, respectively. Weekly each load bus profile used in this study is shown in Figure 6. The BESS is turned off temporarily when it achieves either upper or lower limits. In this work, since the voltage profiles at all times are above the minimum limit of 0.95 pu, the BESS does not discharge when the PVDG is active. Therefore the BESS is set to discharge at 7 pm, right after the PVDG is inactive at the night time in order to provide a capacity for the BESS to continue charging on the following day.

**Figure 7:**
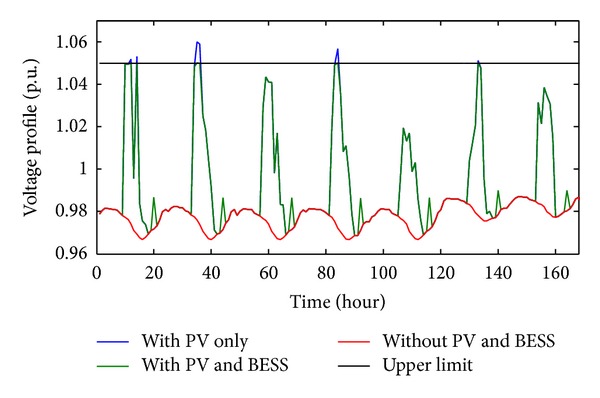
Effect of PV and BESS on PVDG bus voltage profiles.

Figure 7 shows the comparison of voltage profiles of PVDG bus for one week where 3 cases are included, namely, the system without PVDG and BESS, system with PVDG only, and system with PVDG and EOFA optimized BESS. From Figure 7, it can be seen that the voltage rises greatly after the PVDG is installed into the system and exceeds the limit of 1.05 pu. However, the voltage magnitude after the installation of BESS is limited within the maximum limit of 1.05 pu. All system modeling and simulations in this study are done using MATLAB software and distribution load flow program adopted from [35, 36].

Besides, BESS optimized with GSA and FA is included in this work to validate the effectiveness of EOFA in BESS sizing. For the first optimization process in getting the optimal BESS output power for each hour, the maximum iteration number and the population size, *n* in all algorithms, are set to be 50 and 10, respectively, while, for the second optimization process in obtaining the optimal BESS size, those parameters are set to be 100 and 50, respectively, for three algorithms, namely, EOFA, GSA, and FA.

**Figure 8:**
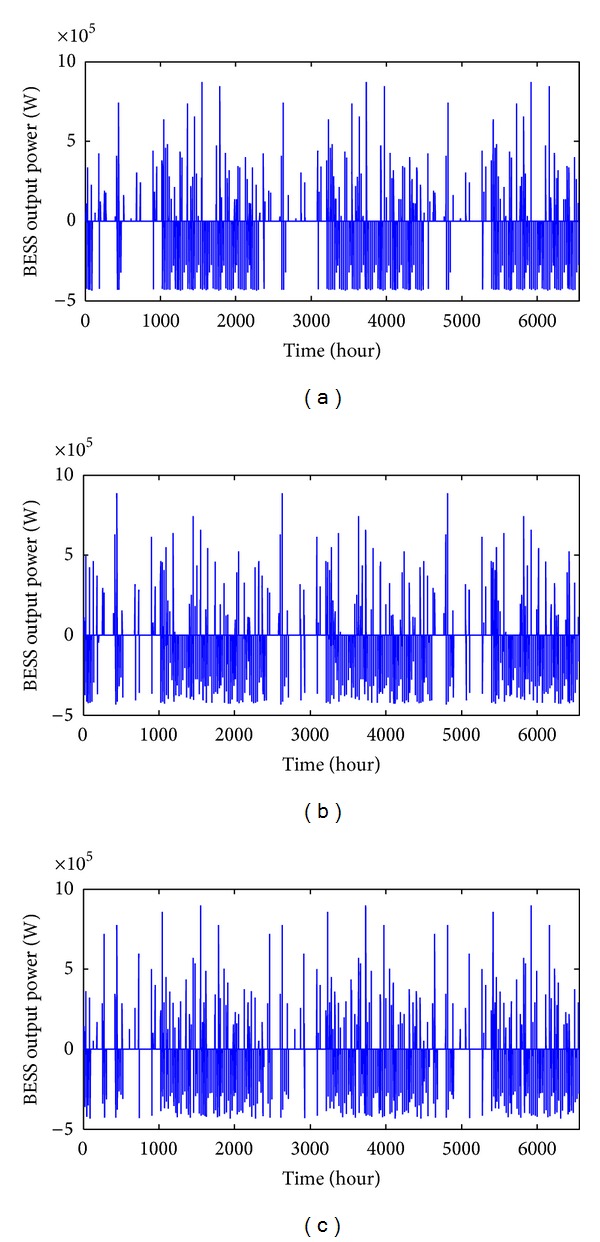
Hourly BESS output power for optimal BESS size obtained with (a) EOFA, (b) FA, and (c) GSA.

**Figure 9:**
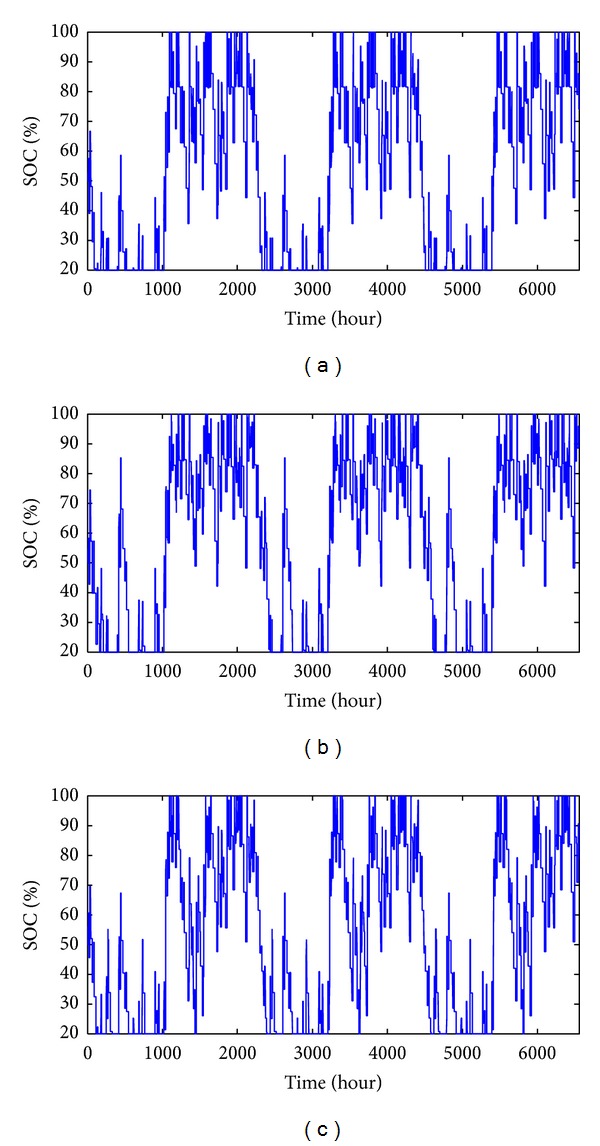
SOC for optimal BESS size obtained with (a) EOFA, (b) FA, and (c) GSA.

The performances of EOFA, FA, and GSA in obtaining the optimal BESS size are discussed as follows.  Figure 8 shows the hourly BESS output power that is to be injected (negative value) or sink (positive value) at Bus 61 in the 69-bus system according to the SOC of optimal BESS size obtained from all three algorithms. The SOC for EOFA, FA, and GSA are illustrated in Figure 9. For EOFA, the BESS was turned off due to the SOC constraint for a total of 385 hours with the optimal BESS capacity of 2.31 MWh. Meanwhile, for FA and GSA, the BESS was turned off due to the SOC constraint for a total of 336 hours and 287 hours with the optimal BESS capacity of 2.42 MWh and 2.39 MWh, respectively. From the result, it can be seen that, by using EOFA, the BESS size is the smallest even though the number of total off-time for the BESS is relatively large. Smaller BESS size is better in terms of saving the installation cost. However, the total number of BESS off-time can be decreased by increasing the BESS size as suggested by GSA and FA algorithm.

**Figure 10:**
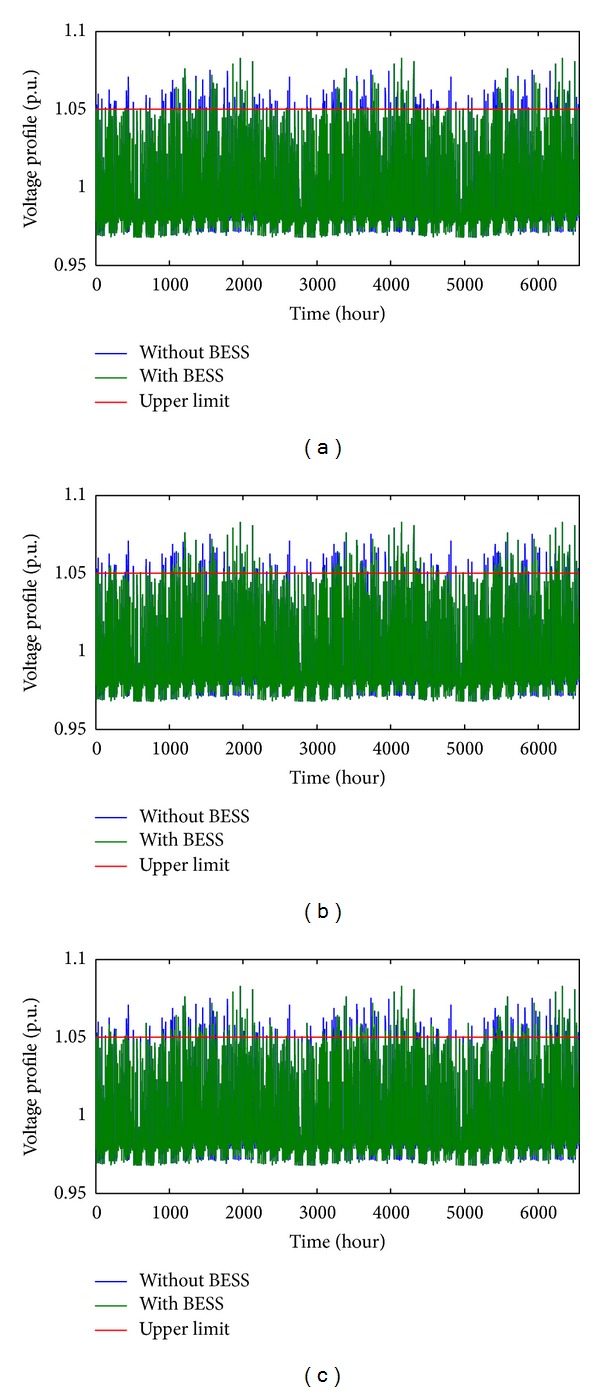
Comparison of voltage profile with and without optimal BESS size obtained with (a) EOFA, (b) FA, and (c) GSA.

Figure 10 shows the comparison of the voltage profile at the PVDG bus with and without BESS for the whole 91 days (6552 hours). In this study, the voltage range is aimed at being between 1.05 pu and 0.95 pu, where, before the BESS was installed, the range falls between 1.08 pu and 0.96 pu. This means that the only voltage rise problem existed in this case. After installing BESS with optimal size obtained with various algorithms, the voltage rises are found to be reduced to the targeted range in most of the time. EOFA keeps most of the voltage values within the range where the voltage values exceed 1.05 pu for a total of 78 hours out of 6552 hours (1.19%). On the other hand, the total number of hours for the voltage values exceeding 1.05 pu for FA and GSA optimized BESS size is 196 hours (2.99%) and 168 hours (2.56%), respectively. It can be seen that EOFA has the best performance comparatively in solving voltage rise problem in the PVDG integrated 69-bus system.  Table 3 shows the summary and comparative results obtained from GSA, FA, and EOFA.

## 6. Conclusion

A new optimization technique named EOFA is presented for determining optimal BESS sizing in order to solve the problem of voltage rise due to the PVDG installation in power distribution systems. The performance and effectiveness of EOFA were extensively tested on 15 unconstrained global optimization functions and the results were compared with other existing optimization techniques, namely, FA and GSA. It can be concluded that the EOFA is more effective than the aforementioned optimization techniques in obtaining the global optimum value for the test functions. The optimization problem formulation aims to reduce the voltage deviation of the system with optimal BESS size. This method was extensively tested on the 69-bus system and the results were compared with FA and GSA. Based on the results, it can be concluded that EOFA is more effective than the FA and GSA in obtaining optimal size for the BESS where EOFA gives the minimum BESS size of 2.39 MWh and minimum number of hours for the voltage values exceeding 1.05 pu which is 78 hours.

## Figures and Tables

**Table 1:** Test functions for unconstrained global optimization.

Function	Name of the function	Dimension size	Global minima
F1	Ackley function	30	0
F2	Beale function	2	0
F3	Bohachevsky function 1	2	0
F4	Bohachevsky function 3	2	0
F5	Griewank function	30	0
F6	Matya function	2	0
F7	Michalewicz function	10	−9.66
F8	Perm function	30	0
F9	Powell function	30	0
F10	Rastrigin function	30	0
F11	Rosenbrock function	30	0
F12	Schwefel function	30	0
F13	Sphere function	30	0
F14	Sum square function	30	0
F15	Zakharov function	30	0

**Table 2:** Comparison of performances for GSA, FA, and EOFA.

Function	Optimization algorithm								
	GSA			FA			EOFA		
	Optimized fitness value								
	Best	Average	Worst	Best	Average	Worst	Best	Average	Worst
F1	0.0096	0.015	0.024	18.52	19.60	19.97∗∗	8.88*E* − 16*	3.8*E* − 15	7.99*E* − 15
F2	2.07*E* − 07*	6.09*E* − 06	6.84*E* − 05	3.60*E* − 06	0.069	0.91∗∗	5.46*E* − 06	3.99*E* − 04	0.0014
F3	1.09*E* − 06	2.06*E* − 05	1.05*E* − 04	0.00021	0.55	3.35∗∗	0∗	4.88*E* − 17	2.22*E* − 16
F4	1.87*E* − 07	9.87*E* − 06	3.98*E* − 05	6.11*E* − 05	0.30	2.08∗∗	0∗	1.78*E* − 17	5.55*E* − 17
F5	6.98*E* − 06	0.0014	0.030	446.74	592.46	686.12∗∗	0∗	2.26*E* − 16	2.78*E* − 15
F6	4.73*E* − 09	1.35*E* − 07	9.11*E* − 07	1.30*E* − 05	0.043	0.58∗∗	1.59*E* − 40*	1.45*E* − 36	8.06*E* − 36
F7	−9.46∗	−8.81	−7.73	−6.34	−4.11	−2.55∗∗	−9.33	−8.90	−7.85
F8	1.41*E* + 82	1.62*E* + 85	8.89*E* + 85**	4.22*E* + 81	2.24*E* + 84	2.52*E* + 85	5.15*E* + 77*	7.42*E* + 80	1.19*E* + 82
F9	0.0014	0.0052	0.012	3658.18	5851.00	9794.40∗∗	9.69*E* − 35*	6.14*E* − 32	7.94*E* − 31
F10	15.99	34.50	53.79	353.45	394.46	429.40∗∗	0∗	0.99	3.19
F11	25.75∗	27.53	29.47	710546.20	1211549	1629028∗∗	28.00	28.73	28.94
F12	8389.22	9719.90	10278.85	8981.36	10257.49	11111.71∗∗	656.6∗	1094.737	1624.44
F13	1.80*E* − 4	3.26*E* − 4	6.04*E* − 4	111.90	139.60	156.75∗∗	2.31*E* − 35*	1.06*E* − 32	5.13*E* − 32
F14	0.0019	0.0048	0.011	6228.04	8787.32	10279.92∗∗	3.52*E* − 34*	1.46*E* − 31	6.56*E* − 31
F15	24.16	51.79	73.96	708.22	5.47*E* + 08	3.92*E* + 09**	5.84*E* − 35*	1.92*E* − 30	1.60*E* − 29

**Table 3:** Comparison of performance for GSA, FA, and EOFA in battery sizing.

Optimization algorithm	PV size (MWp)	Maximum load (MW)	Minimum load (MW)	BESS capacity (MWh)	BESS off- time (hour)	Total number of hours the voltage exceeding 1.05 pu	
						With BESS (hour)	Without BESS (hour)
GSA	3.66	1.52	0.61	2.39	287	168	297
FA				2.42	336	196	297
EOFA				2.31	385	78	297
